# Differences in gastric microbiota and mucosal function between patients with chronic superficial gastritis and intestinal metaplasia

**DOI:** 10.3389/fmicb.2022.950325

**Published:** 2022-11-17

**Authors:** Yingxia Li, Libin Jiang, Zhichao Li, Yali Liu, Bo Xiao, Yan Ding, Hongtao Wen

**Affiliations:** ^1^Department of Gastroenterology, The First Affiliated Hospital of Zhengzhou University, Zhengzhou, China; ^2^College of Animal Science and Technology, Henan Agricultural University, Zhengzhou, China

**Keywords:** gastric cancer, chronic superficial gastritis, intestinal metaplasia, mucin, gastrointestinal microbiota, 16S rRNA gene

## Abstract

Chronic superficial gastritis (CSG) and intestinal metaplasia (IM) can further develop into gastric cancer, which seriously endangers the health of people all over the world. In this study, the differences in gastric microbiota between CSG patients and IM patients were detected by 16S rRNA gene sequencing. As the expression levels of mucin and CDX2 are closely related to IM, the expression differences of mucin (MUC2 and MUC5AC) and CDX2 in the gastric mucosa of CSG patients and IM patients were detected by Western blot and qRT-PCR. The results showed that both Faith_pd and Observed_species indexes of microbiota in the gastric juice of CSG patients were significantly higher than those of IM patients. At the genus level, *Thermus* and *Anoxybacillus* were dominant in the gastric juice of IM patients, and *Helicobacter* was dominant in the gastric juice of CSG patients. Non-metric multidimensional scaling (NMDS) demonstrated that the dispersion of samples in the CSG group is greater than that in the IM group, and some samples in the CSG group are clustered with samples in the IM group. The KEGG metabolic pathway difference analysis of gastric juice microbiota in CSG and IM patients revealed that the gastric juice microbiota in the CSG and IM patients were significantly enriched in the amino acid metabolism, carbohydrate metabolism, and metabolism of cofactors and vitamins, and the functional differences between the two groups were mainly concentrated in the bacterial secretion system (VirB1, VirB2, VirB3, VirD2, and VirD4). In conclusion, there are significant differences in gastric microbiota and mucosal function between the CSG and IM patients. Moreover, the results of this study may provide a new means for the detection of CSG and IM and a new direction for the prevention and treatment of CSG and IM.

## Introduction

At present, gastric cancer (GC) is one of the major fatal tumors in the world (Ferlay et al., 2015); especially in men in underdeveloped countries, the incidence rate and mortality of GC are among the highest (Patru et al., 2013; Joliat et al., 2015; Wong et al., 2021). A large number of studies have shown that GC is a complex disease caused by the interaction of environmental and host-related factors (Stoicov et al., 2004; Huang et al., 2012; Gao et al., 2016). The key factors leading to the high mortality of GC are the lack of symptoms of early GC, the clinical manifestations of late GC, and the potential biological and genetic heterogeneity (Yamamoto et al., 2014; Kim et al., 2019). Chronic gastritis and intestinal metaplasia (IM) are considered to be important steps in the pathogenesis of GC (Spence et al., 2017). Among them, IM is a recognized precancerous lesion of GC, and patients with IM can increase the risk of GC (El-Zimaity et al., 2001). In IM, it is estimated that 0.13–0.25% of patients have the risk of GC every year (De Vries et al., 2008).

Gastric mucosal IM is a pathological process in which intestinal epithelial cells replace gastric mucosal epithelial cells, that is, there are epithelial cells similar to the intestinal mucosa in the gastric mucosa (Abdelfattah et al., 2021; Jonaitis et al., 2021; Zhang et al., 2021; He et al., 2022). IM is common in the stomach and esophagus, but can also be seen in the gallbladder, bile duct, uterus, bladder, pelvis, ureter, and urethra (Deniz et al., 2010; Rubio et al., 2016). IM is a process of quantitative change. Qualitative change is an important hint of precancerous lesions of gastric cancer (PLGC). IM has been regarded as a PLGC, and the diagnosis is based on gastroscopy and histopathological results (Butov et al., 1987; Malik et al., 2017; Jencks et al., 2018; Li et al., 2019; Al Hennawi et al., 2021). The recognized occurrence and development model of GC is the model proposed by Correa (1988), and the specific process is as follows: normal gastric mucosa, chronic superficial gastritis, chronic atrophic gastritis, IM of small intestine, IM of large intestine, moderate and severe dysplasia, and GC. GC is one of the most common malignant tumors in the world, and its prevalence is second only to lung cancer; IM is the key to transforming into GC (Malik et al., 2017). An epidemiological survey has confirmed that the family history and personal history of patients with chronic atrophic gastritis are closely related to the incidence of PLGC, especially the personal history of patients with chronic atrophic gastritis, while 36% of patients with chronic superficial gastritis are transformed into IM and 1% into GC (Wu et al., 2013). It can be seen that the prevention and treatment of CSG and IM are of great significance for the prevention of GC.

The international agency for research on cancer already classified *Helicobacter pylori* as a first-class carcinogen, which is closely related to about 75% of the new cases of GC in the world every year (Grochowska et al., 2022). *H. pylori* causes an inflammatory immune response in gastric mucosa and destroys the gastric mucosal barrier (Radziejewska, 2012; Oluwole, 2015). The results of long-term follow-up observation of relevant studies suggest that the longer the continuous infection time of *H. pylori*, the heavier the degree of gastric mucosal inflammation, the higher the incidence and severity of IM, which is the main factor promoting gastric mucosal atrophy and is an important cause of IM (Yakirevich and Resnick, 2013; Ben Slama et al., 2016; Tsukamoto et al., 2017; Hwang et al., 2018). The virulence genes of *H. pylori* include cytotoxin-associated gene A (CagA), specific vacuolar toxin A gene (VacA), blood group antigen-binding adhesin A gene (BabA), induced expression factor after gastric epithelial contact (IceA), and sialic acid-binding adhesin gene (SabA), and CagA and VacA genes are more closely related to GC (Boyanova et al., 2010; Shiota et al., 2010; Abadi et al., 2013). It has been reported abroad that CagA-positive *H. pylori* infection can significantly aggravate the inflammatory reaction of gastric mucosa and accelerate gastric mucosal atrophy and IM (Abdo-Francis et al., 2010; Abe et al., 2011). Some scholars also proposed that the important mechanism of *H. pylori*-induced gastric mucosal injury is that it can release and stimulate vascular endothelial cells, macrophages, and epithelial cells to produce inflammatory chemokines and cause an inflammatory immune response among lymphocytes, macrophages, and neutrophils in the gastric mucosa (Wroblewski and Peek, 2016).

Although the theory of intestinal adenoid glands replacing gastric inherent glands is currently recognized by most researchers, *H. pylori* infection causes damage to gastric mucosa (chronic superficial gastritis, CSG), and the pathological process from CSG to IM has not been fully clarified, especially the changes in the structure and function of microbiota in the stomach (Zhang et al., 2005). After all, gastrointestinal microorganisms are closely related to the growth, development, and health of the host. Therefore, in this study, we identified the differences in gastric juice microbiota between CSG and IM patients by Illumina MiSeq sequencing, in order to analyze the core microbiota and function of CSG-transformed IM, provide reference for the prevention and treatment of IM, and prevent further deterioration of IM.

## Materials and methods

### Case source

The CSG and IM patients who met the inclusion criteria in the gastroenterology clinic of the First Affiliated Hospital of Zhengzhou University were selected and the sample size *N*_CSG_ = 12 and *N*_IM_ = 12 were obtained. The ages ranged from 33 to 76 years, with an average of CSG: 66.0 ± 7.0 years and IM: 60.5 ± 11.2 years. The patients were required to be aged over 30 years, have no contraindications of gastroscopy, and have no known severe heart, lung, and other important organ dysfunctions.

### Ethical review

The clinical research plan met the ethical standards and was approved by the clinical ethics committee of the scientific research project of the First Affiliated Hospital of Zhengzhou University (2021L03521).

### Sample collection

FUJINENG EG-485ZH endoscope was used to observe and sample the antral tissue and gastric juices after routine gastroscopy (Alcohol, spicy food, antiplatelet drug aspirin, and clopidogrel are prohibited 7 days before gastroscopy). The antral tissue was stained with hematoxylin and eosin (H&E), and the expressions of CDX2, MUC2, and MUC5AC genes and proteins were detected by qRT-PCR and Western blot, respectively. The primer sequences of qRT-PCR are shown in Table 1. In addition, 16S rRNA gene sequencing of gastric juices was performed using Illumina MiSeq, and the procedure is as follows.

**Table 1:** Primers for qRT-PCR.

**Name**	**Gene ID**	**Forward**	**Reverse**	**Length**
*MUC2*	4583	GAGGGCAGAACCCGAAACC	GGCGAAGTTGTAGTCGCAGAG	117
*CDX2*	1045	GACGTGAGCATGTACCCTAGC	GCGTAGCCATTCCAGTCCT	215
*MUC5AC*	4568	AGTGTCCCCCATGCACTGA	ACACCCTCCACAAGAAAGCG	165

### Extraction and database construction of gastric juices genomic DNA

Total genomic DNA samples of bacteria in gastric juices were extracted using DNA Extraction Kit (MP Biomedical, Santa Ana, CA, USA) according to the manufacturer's instructions and stored at −20°C before further analysis. The concentration and quality of extracted DNA were measured by NanoDrop ND-1000 spectrophotometer and agarose gel electrophoresis, respectively. The V3–V4 region of the bacterial 16S rRNA gene was amplified by PCR using forward primer (5′-ACTCCTACGGGGAGGGCAGCA-3′) and reverse primer (5′-GGACTACHVGGTWTCTAAT-3′). Illumina's TruSeq Nano DNA LT Library Prep Kit was used to prepare the sequencing library.

### Bioinformatics analysis

#### Alpha- and beta-diversity analysis

In this study, alpha-diversity index and beta-diversity index were used to characterize the diversity of species within and between habitats, respectively, so as to comprehensively evaluate their overall diversity. *Alpha diversity*: Using QIIME 2 (2019.4) and ggplot2 package in R language, alpha-diversity index (mainly including Chao1, observed species, Shannon, Simpson and Faith's PD) was calculated according to the ASV/OTU table that is not flattened to detect biological diversity. *Beta diversity*: The uclust function of the stat package in R language and the UPGMA algorithm (i.e., average clustering method) were used for the clustering analysis of Bray Curtis distance matrix by default, and the R script ggtree package was used for visualization. The clustering effect was measured by the branch length of the clustering tree.

#### Species composition analysis

The “qiime taxa barplot” command in QIIME2 was coined, statistics on the feature table after removing singleton were made, the visualization of the composition distribution of each sample at the genus level was realized, and the analysis results in a histogram were presented. According to the results of the taxonomic annotation of sequence species and the selected samples, the number of taxons contained at the genus level in the species annotation results of these samples were counted. An interactive display of community taxonomic composition was observed using the Krona software (https://github.com/marbl/Krona/wiki).

#### Species difference and standard species analysis

After exploring the differences in microbial community composition, we also need to know which species mainly cause these differences. The LEfSe package in Python was used to draw the histogram of the LDA value distribution of significantly different species to show the significantly enriched species in each group (note that there is no significant downregulation) and their importance.

#### Functional potential prediction

The obtained functional units can obtain the abundance value of the metabolic pathway according to the metabolic pathway database and R language analysis. Then, using the normalized functional unit abundance table, the R script is used to calculate the distance matrix in R and conduct PCoA analysis. The PCoA coordinates of the sample points are the outputs and are drawn into a two-dimensional scatter diagram. Finally, after obtaining the abundance data of metabolic pathways, we tried to find out the metabolic pathways with significant differences between groups.

### Statistical analysis

A completely randomized test design was used in this study. The significance of the difference between the means of the groups was determined by ANOVA or Student's *t*-test. Differences with *P* < 0.05 (^*^) and *P* < 0.01 (^**^) were considered to be significant and extremely significant, respectively. The statistical calculations used in this study were performed using IBM SPSS 24.0.

## Results

### Gastroscopy, H&E staining, and mucosal protein expression in CSG and IM patients

**Figure 1:**
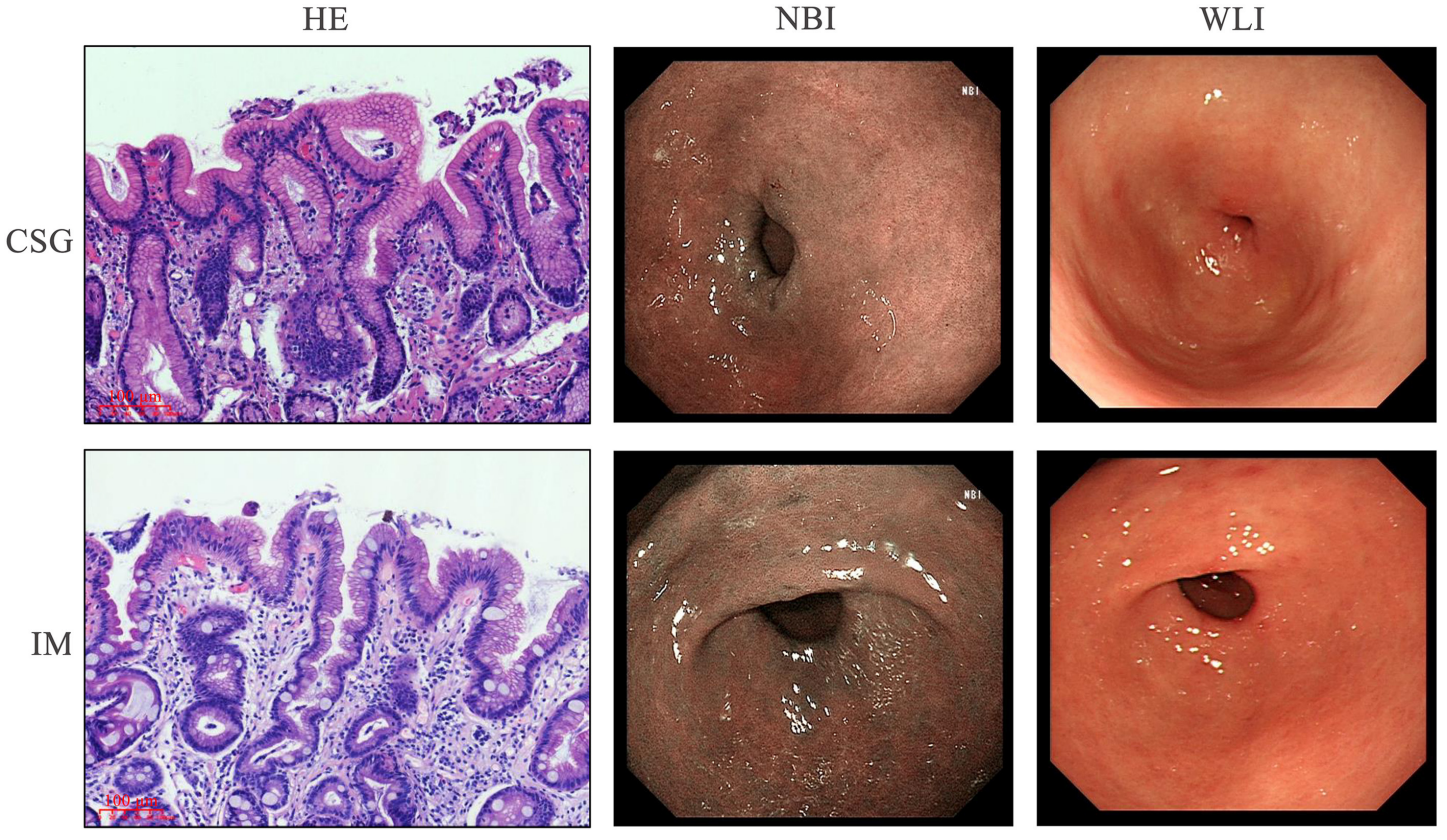
Gastroscopy and histological staining in patients with CSG and IM.

The CSG and IM patients were diagnosed through gastroscopy. Narrow band imaging (NBI) and white light illumination (WLI) microscopic examination indicated that the mucosa of gastric antrum near the pylorus was red in CSG patients, suggesting the existence of inflammation. The mucosa of gastric antra was red and rough, and multiple flat and concave lesions were seen in IM patients, suggesting focal antral IM and inflammation on the surface epithelium and glandular neck of the gastric antrum. The clinical characteristics of CSG and IM patients are shown in Table 2. The morphology of metaplasia sites in the gastric mucosa was determined, and then the tissue structure and microbiota structure of these sites were further described in detail. H&E staining revealed that CSG patients had inflammation of the gastric antrum mucosa. IM patients had IM characterized by goblet cells and accompanied by inflammation (Figure 1).

**Table 2:** The clinical characteristics of the CSG and IM patients.

**Clinical characteristics**	**CSG group (*n* = 12)**	**IM group (*n* = 12)**	***P*-value**
**Age**			0.098
< 55	7	3	
≥55	5	9	
**Gender**			0.673
Male	7	8	
Female	5	4	
**Distribution in stomach**			0.016
Cardia and fundus of stomach	2	1	
Greater curvature of gastric body	1	1	
Lesser curvature side of gastric body	0	2	
Anterior wall of gastric body	0	0	
Posterior wall of gastric body	0	1	
Antrum gastricum	9	7	
**Severity**			0.043
Mild to moderate	5	6	
Moderate	4	4	
Severe	3	2	
**Relationship with atrophy and atypical hyperplasia**			Not applicable
Simple intestinal metaplasia	10	8	
With atrophy of gastric mucosa	2	4	
Associated with atypical hyperplasia	0	0	
**Endoscopy description**			0.558
Redness of mucous membrane	6	3	
Surface erosion	3	2	
Ulcer	0	2	
Variola-like protuberance	1	2	
Accompanied by ≥2	1	1	
**Hp detection**			0.041
Postive	10	4	
Negative	2	8	

### Expression levels of CDX2, MUC2, and MUC5AC

**Figure 2:**
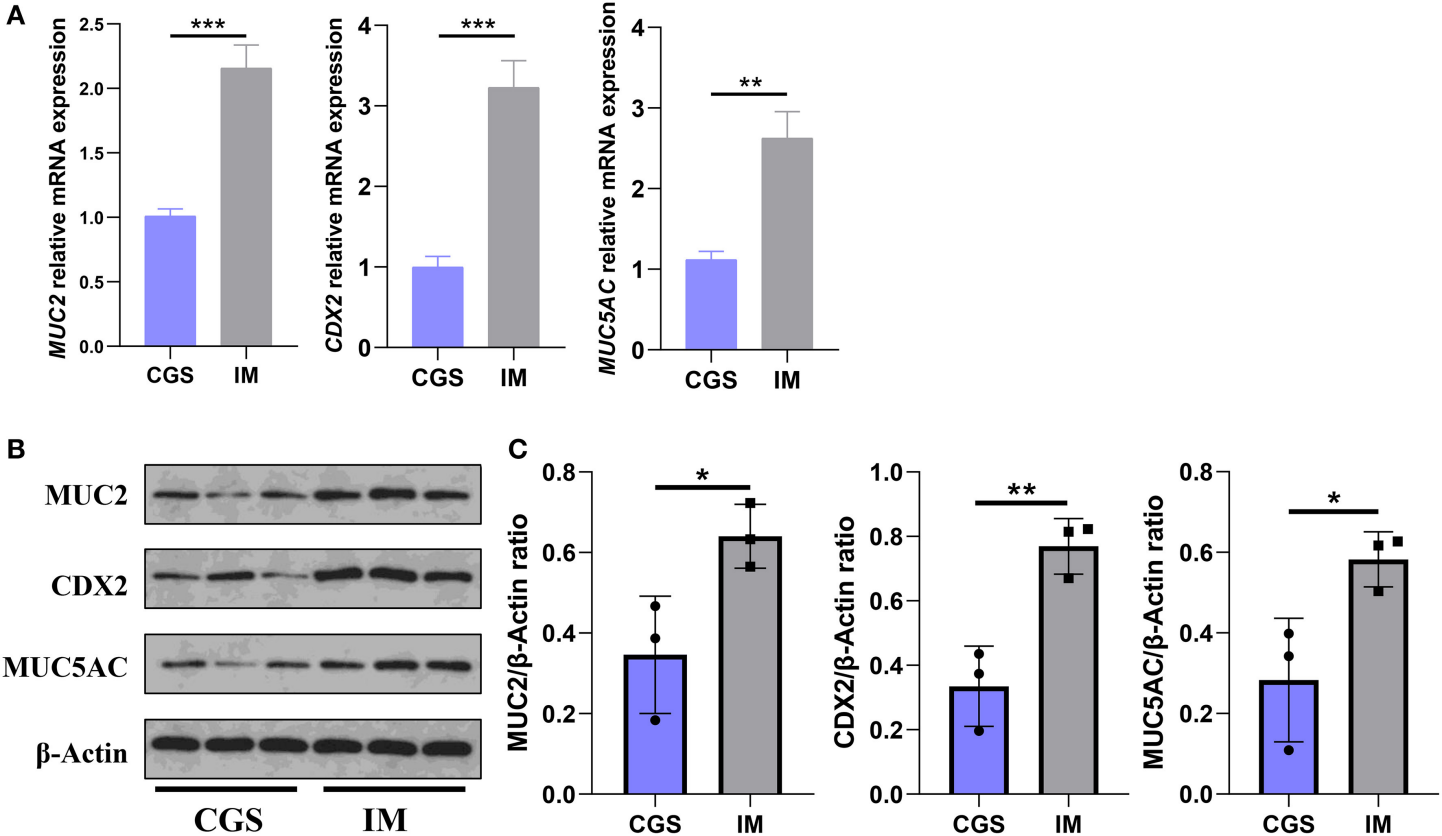
Differences in the expression of MUC2, MUC5AC, and CDX2 in gastric antral mucosa of patients with CSG and IM. **(A)** qRT-PCR results of *MUC2, MUC5AC*, and *CDX2* in Gaussian antral mucosa of patients with CSG and IM. **(B,C)** Western blot results of MUC2, MUC5AC, and CDX2 in Gaussian antral mucosa of patients with CSG and IM. Asterisks denote significance (*p < 0.05; **p < 0.01; ***p < 0.001).

The results of qRT-PCR and Western blot demonstrated that the expressions of MUC2, MUC5AC, and CDX2 in gastric antrum mucosa of IM patients were higher than those of CSG patients, and the difference was statistically significant (Figure 2).

### Pepsinogen and alpha-diversity analysis

From the age of patients, it was found that the average age of IM patients was lower than that of CSG patients, and the difference was statistically significant (*P* < 0.05). The results of the pepsinogen test confirmed that the levels of pepsinogen I, II and III in IM patients were lower than that in CSG patients, and the difference was statistically significant (*P* < 0.01 or *P* < 0.001). The results of alpha diversity of microbiota indicated that the Faith_pd and Observed_species indexes of IM patients were significantly lower than that of CSG patients (*P* < 0.05), while the Chao1, Shannon, and Simpson indexes between the two groups had no significant difference (*P* > 0.0; Table 3).

**Table 3:** Baseline patient characteristics and microbiome reads of samples.

**Variable**	**CSG**	**IM**	***P*-value**
*N*	12	12	
Age, year, mean ± SD	66.0 ± 7.0	60.5 ± 11.2	0.0324^*^
Male, *n* (%)	7 (58.3%)	8 (66.7%)	
**Pepsinogen test**			
Pepsinogen I, ng/mL mean ± SD	96.4 ± 22.4	51.2 ± 12.4	< 0.001^***^
Pepsinogen II, ng/mL mean ± SD	28.1 ± 8.1	18.7 ± 5.8	0.0034^**^
Pepsinogen III, ng/mL mean ± SD	7.0 ± 2.4	2.9 ± 1.1	< 0.001^***^
**Microbiome reads, mean ± SD**			
Read count	105,157 ± 31139	98,513 ± 22293	0.5540
ASVs	97,665 ± 27,115	92,028 ± 20,776	0.5734
Chao1	892.7 ± 302.5	773.7 ± 130.7	0.2240
Shannon's diversity index	3.78 ± 0.99	3.94 ± 0.78	0.6776
Simpson's diversity index	0.72 ± 0.13	0.77 ± 0.08	0.2523
Faith_pd	441.5 ± 175.4	281.7 ± 90.2	0.0102^*^
Observed_species	884.7 ± 261.5	698.9 ± 138.3	0.0407^*^

Asterisks denote significance (

* p < 0.05;

** p < 0.01;

*** p < 0.001).

### Species composition and standard species analysis

**Figure 3:**
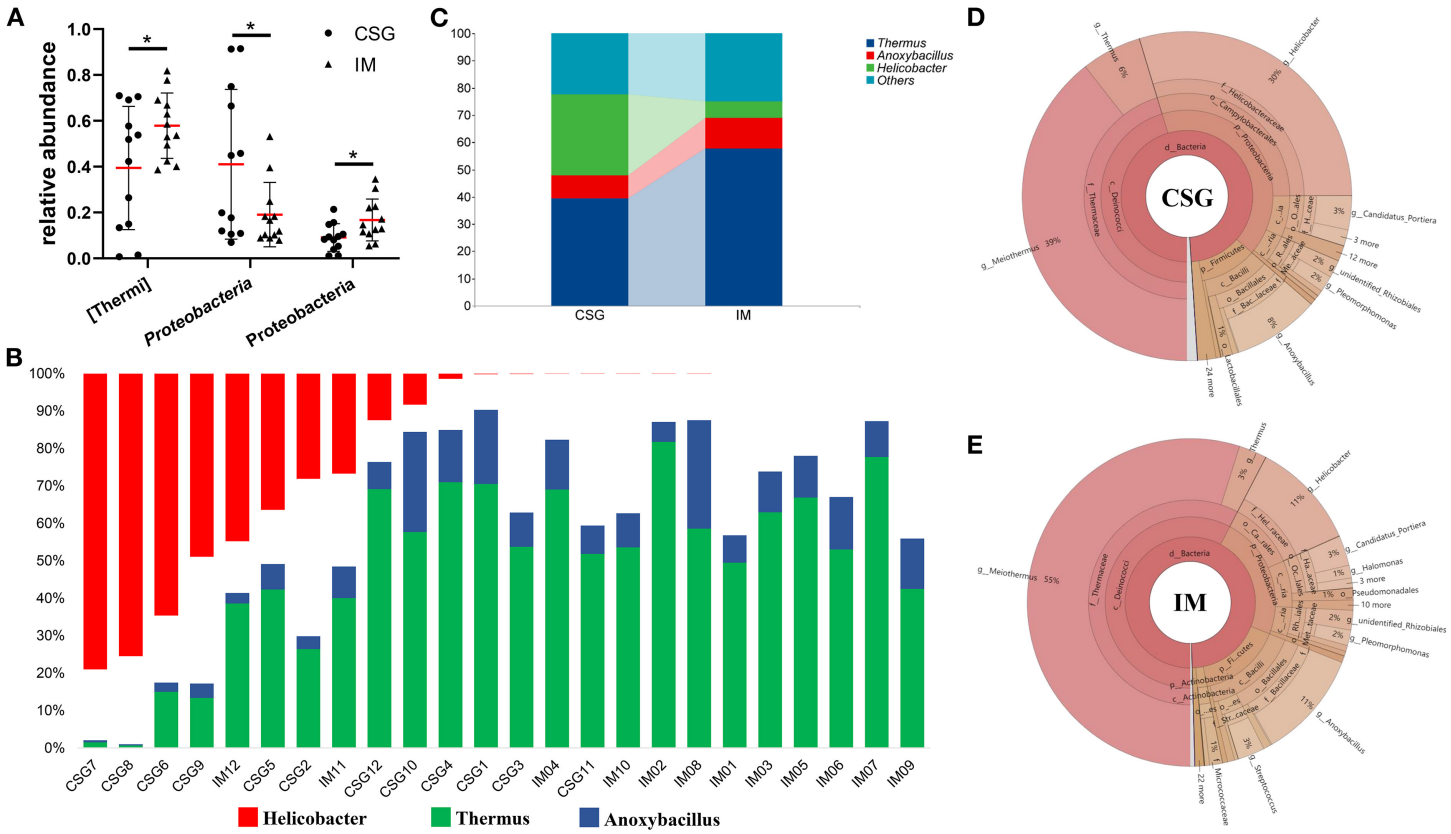
Differences in microbiota composition of gastric juices between CSG and IM patients. **(A)** Composition difference of gastric juices microbiota at the phylum level between CSG and IM patients. **(B)** Composition difference of gastric juices microbiota at the genus level between CSG and IM patients. **(C)** An integrated representation of the differences in the composition of gastric microbiota between CSG and IM patients at the genus level. **(D,E)** Krona species composition of gastric juices microbiota in patients with CSG and IM. Asterisks denote significance (*p < 0.05).

**Figure 4:**
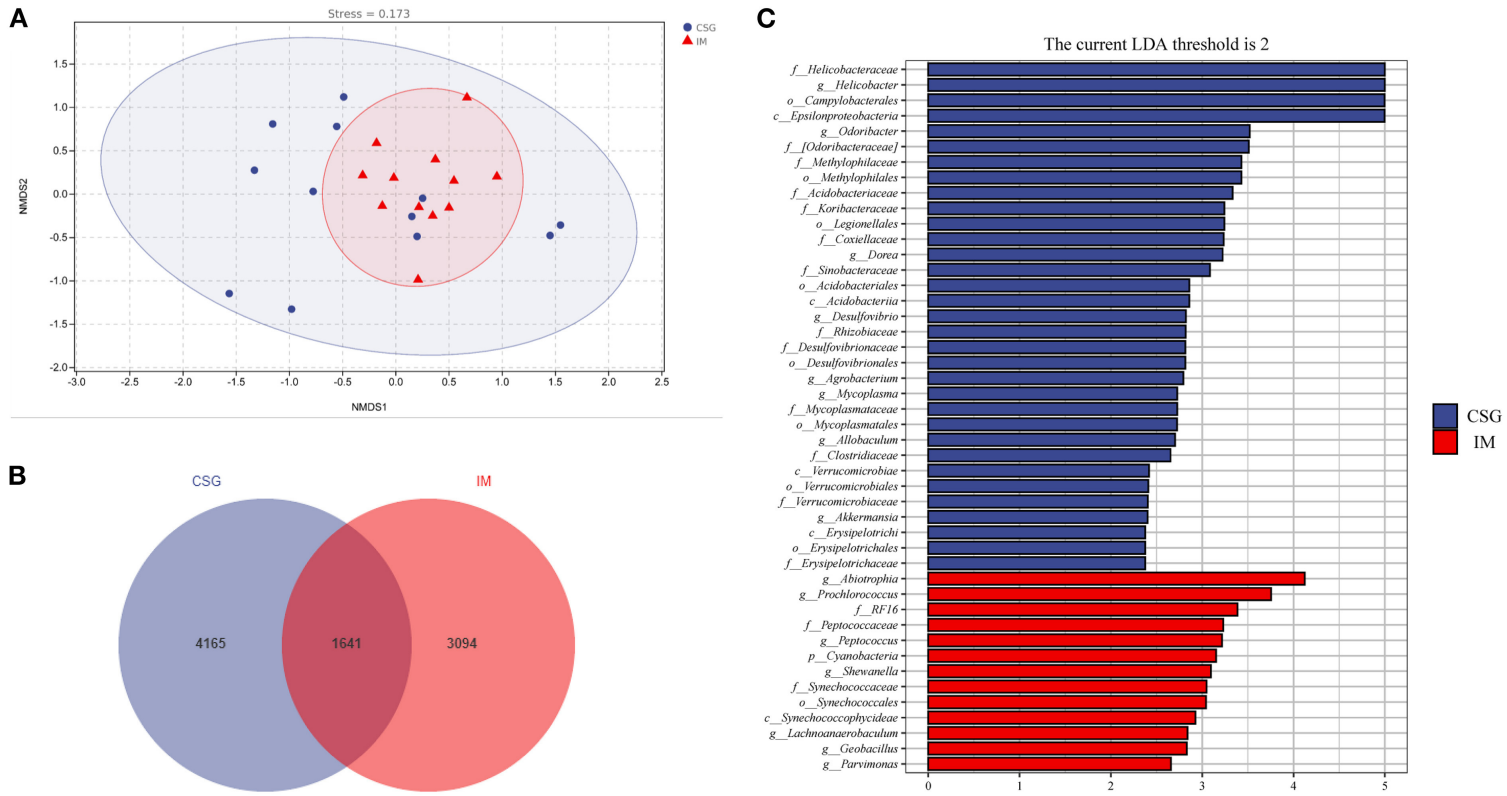
Species differences and marker species analysis of gastric juices microbiota in patients with CSG and IM. **(A)** NMDS analysis. **(B)** ASV/OTU Venn diagram. **(C)** LEfSe analysis.

The relative abundances of the top three with the most significant differences at the phylum and genus levels were statistically analyzed. At the phylum level, the relative abundances of Thermus and Firmicutes in IM patients were significantly higher than that in CSG patients, while the relative abundance of Proteobacteria in IM patients was significantly lower than that in CSG patients (Figures 3A–C). At the genus level, *Thermus* and *Anoxybacillus* were dominant in IM patients; some IM patients also had *Helicobacte*r, while the *Helicobacter* was dominant in CSG patients; and some CSG patients had both *Thermus* and *Anoxybacillus* (Figure 3D). The krona circle chart represents seven classification levels, namely, domain, phylum, class, order, family, genus, and species, from inside to outside. The size of the sector reflects the relative abundance of different taxons. The results revealed that at the phylum level, Firmicutes and Proteobacteria were dominant in both IM and CSG patients, and the value of Proteobacteria/Firmicutes in CSG patients was higher than that in IM patients. At the genus level, *Meiothermus, Helicobacter, Anoxybacillus*, and *Thermus* were dominant in IM patients, and the relative abundance of *Meiothermus* in IM patients (55%) was higher than that in CSG patients (39%) (Figures 3E,F). NMDS results show that there are great differences in sample distribution between the IM group and the CSG group. In short, the dispersion of samples in the IM group is less than that in the CSG group, and some samples in the CSG group are clustered with samples in the IM group (Figure 4A). Venn diagram shows that there are 4,165 and 3,094 ASV/OUT in the CSG group and IM group, respectively, and shows that the number of duplicate ASV/OUT in CSG group and IM group was 1641 (Figure 4B). Lefse analysis showed that the first three microbial species with significant differences in CSG patients were *f_Helicobacteraceae, g_Helicobacter*, and *o_Campylobacterales*, respectively. The first three microbial species with significant differences in IM patients were *g_Abiotrophia, g_Synechococcus*, and *g_Ralstonia*, respectively (Figure 4C).

### Metabolic pathway statistics

**Figure 5:**
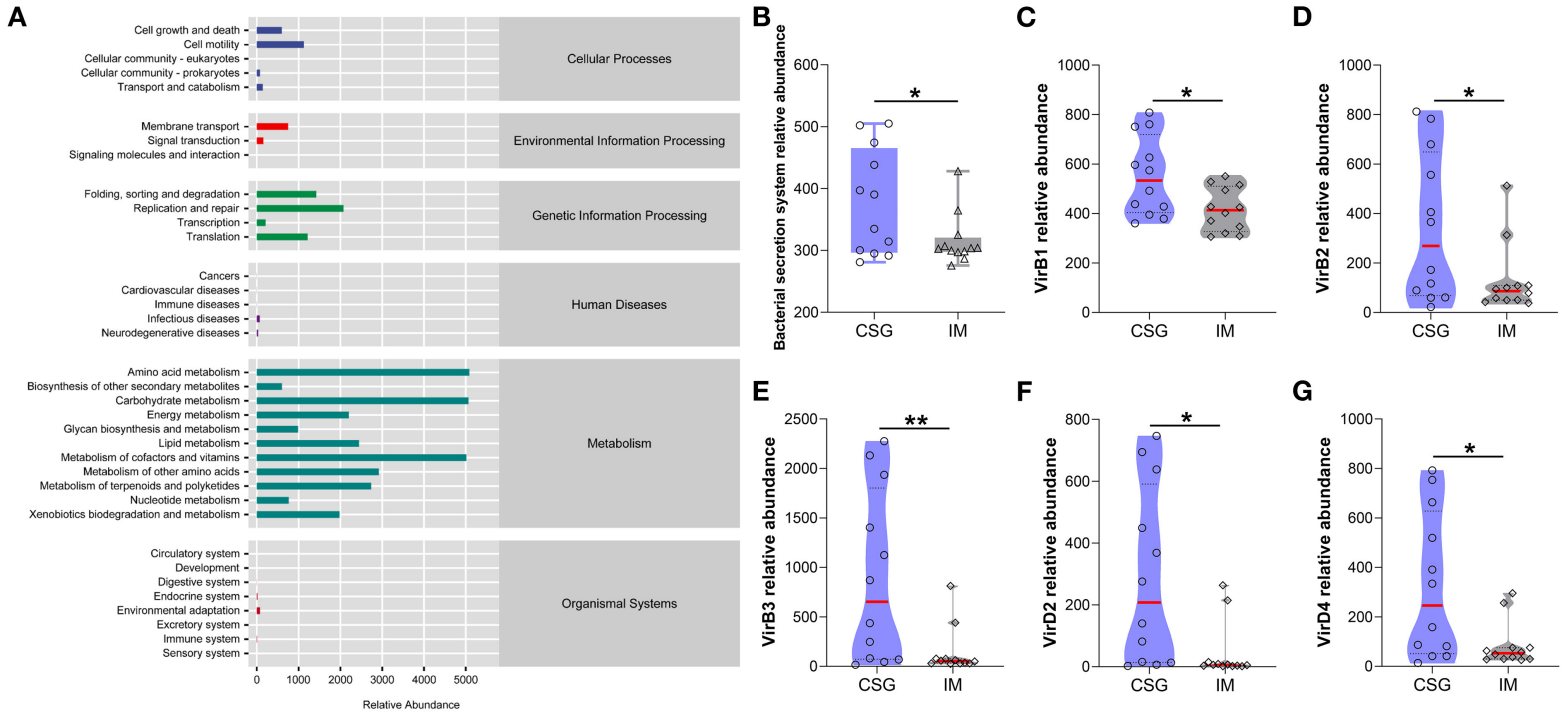
Analysis of KEGG metabolic pathway difference of gastric juices microbiota in patients with CSG and IM. **(A)** Total KEGG metabolic pathway. **(B)** Relative abundance of bacterial secretion system between the gastric juice microbiota of CSG and IM patients. **(C)** VirB1 relative abundance between the gastric juice microbiota of CSG and IM patients. **(D)** VirB2 relative abundance between the gastric juice microbiota of CSG and IM patients. **(E)** VirB3 relative abundance between the gastric juice microbiota of CSG and IM patients. **(F)** VirD2 relative abundance between the gastric juice microbiota of CSG and IM patients. **(G)** VirD4 relative abundance between the gastric juice microbiota of CSG and IM patients. Asterisks denote significance (*p < 0.05; **p < 0.01).

The KEGG metabolic pathway results demonstrated that most microorganisms were mainly involved in metabolic pathways, including amino acid metabolism, carbohydrate metabolism, and cofactor and vitamin metabolism (Figure 5A). Further analysis of differential metabolic pathways revealed that the relative abundance of bacterial secretory system, VirB1, VirB2, VirB3, VirD2, and VirD4 in the gastric juices of CSG patients was significantly higher than that of IM patients (*P* < 0.05) (Figures 5B–G).

## Discussion

Under normal circumstances, there is a balanced state between microorganisms and the host, that is, a steady state, which plays a role in maintaining health (Comito et al., 2014). The invasion of external microbiota and inflammation can promote the homeostasis imbalance between the host and microorganisms, that is, the emergence of a disease or pre-disease state (Antonini et al., 2019). Some scholars believe that taking symbiotic functional bodies as the starting point and integrating various methods to study the interaction mechanism between microbiota and host is an effective means to solve the relationship between host and microorganism and to study and prevent diseases (Kreisinger et al., 2017; Lim and Kim, 2017). Therefore, the purpose of this study was to explore the differences in gastric juice microbiota between CSG and IM patients, analyze the core microbiota and function of CSG transforming IM, and provide reference for preventing and treating IM and preventing IM from further deterioration.

In this study, H&E staining results indicated that IM patients had no microvilli or a few uneven microvilli, which were composed of columnar cells and goblet cells. There was no obvious atypia in the nucleus. The results of the microbiome showed that a higher abundance of *Thermus* and *Anoxybacillus* was detected in the gastric juices of IM patients and some CSG patients with IM, which may be closely related to the occurrence and development of IM. In most CSG patients, the relative abundances of *Thermus* and *Anoxybacillus* were lower, while the relative abundances of *Helicobacter* were higher. *H. pylori* is a gram-negative bacterium and is the most important pathogen in the development of CSG and IM all over the world (Huang et al., 2019). It has been listed as a class of carcinogens by the World Health Organization (Grochowska et al., 2022). The history of *H. pylori* infection is associated with a threefold increase in the risk of non-cardiac GC in a lifetime (Yang et al., 2022). Moreover, *Helicobacter* and *Anoxybacillus* can be detected simultaneously when the two diseases (CSG + IM) exist simultaneously. From the result trend, the relative abundance of microbiota in CSG patients is developing toward that in IM patients. Meanwhile, NBI and WLI results revealed that CSG patients and IM patients had similar gastric lesions, and IM patients may be more serious, which may be consistent with the recognized model of gastric carcinogenesis and development proposed by Correa (1988). To survive, reproduce, and spread in the host, *H. pylori* must secrete some virulence factors with protein properties. To adapt to their living environment, some non-pathogenic bacteria secrete some proteins (Yamaoka, 2010). The system in which bacteria rely on a secretory pathway to transport proteins across cytoplasmic membrane is called the secretory system (Hilbi and Haas, 2012). In this study, the results of the KEGG metabolic pathway confirmed that the bacterial secretory system, VirB1, VirB2, VirB3, VirD2, and VirD4, of CSG patients were significantly higher than that of IM patients (*P* < 0.05). In addition, some studies have confirmed that VirB and VirD proteins are associated with the inflammation of gastric mucosa.

To further explore the impact of microbial differences on the host, we explored the differences in the expression levels of mucin (MUC2 and MUC5AC) and CDX in the host gastric mucosa between CSG patients and IM patients. CDX2 is a member of the tail-type homeobox transcription factor family. It is an intestinal-specific transcription factor, which can directly participate in the regulation of intestinal cell proliferation, differentiation, intestinal development, intestinal phenotype maintenance, and other biological processes. Mucin is a specific product secreted by the mucous epithelium of the digestive tract and is an important component of the digestive tract mucus barrier. MUC2 is a typical mucin secreted by the intestine, and MUC5AC is one of the representatives of mucin secreted by the stomach. In healthy adults, CDX2 and MUC2 are only expressed in the intestinal tract, but not in the gastric mucosa. When IM occurs in the gastric mucosa, the expression of CDX2 and MUC2 can significantly increase.

The results of this study demonstrated that MUC2, CDX2, and MUC5AC were expressed in gastric mucosal cells of the CSG group. MUC2, CDX2, and MUC5AC were also detected in gastric mucosal cells of the IM group, and the difference in expression between the two groups was statistically significant. The activation of CDX2 is also considered to be a key factor in the initiation of IM, which is closely related to the occurrence and development of IM (Barros et al., 2012). There is evidence that ectopic expression of CDX2 can induce normal gastric epithelial cells to differentiate into intestinal epithelial cells (Silberg et al., 2002). CDX2 protein may act on the promoter of the MUC2 gene, thus inducing the expression of gut-specific genes such as MUC2 in gastric epithelial cells and ultimately leading to the transformation of gastric epithelial cells into intestinal epithelial cells. At this stage, all reports prove that CDX2 and MUC2 are highly expressed in IM, and their expression is a significantly positive correlation (Roessler et al., 2005). Mucus is a composite gel composed of mucin glycoprotein, water, immunoglobulin A (IGA), bioactive peptides, and metabolites, which is mainly secreted by goblet cells (Birchenough et al., 2015). Mucus covers the epithelium in two layers: one is the outer loose layer that allows symbiotic microbiota, and the other is the inner tight layer that excludes microbiota (Theodoratou et al., 2014; Bergstrom et al., 2020). This may explain why there are high relative abundances of microbiota in the stomach of CSG and CSG with IM patients. Moreover, *H. pylori* was detected in the gastric juice of some IM patients, which may be related to the abnormal expression of mucin (MUC2) and further develop into IM with gastritis. In CSG patients, in the process of *H. pylori* taking root and growing, the toxins, enzymes, and various cytokines secreted by *H. pylori* can cause gastric mucosal damage, gastric mucosal damage mediated by host immune response, and abnormal gastric acid secretion and then form diseases including gastritis.

## Conclusion

In this study, the difference in gastric microbiota between CSG patients and IM patients was analyzed, and it was found that *Helicobacter, Thermus*, and *Anoxybacillus* were the microbiota of core differences in the stomach of CSG and IM patients, and the metabolic pathway of gastric microbiota KEGG in the bacterial secretion system of the two groups of patients was significantly different. In addition, the expression levels of mucin (MUC2 and MUC5AC) and CDX2 in gastric mucosa of CSG and IM patients were also significantly different. The results of this study can provide a new means for the detection of CSG and IM and a new direction for the prevention and treatment of CSG and IM.

## Data availability statement

The datasets presented in this study can be found in online repositories. The names of the repository/repositories and accession number(s) can be found below: https://www.ncbi.nlm.nih.gov/, PRJNA806642.

## Ethics statement

The studies involving human participants were reviewed and approved by Ethics Committee of the First Affiliated Hospital of Zhengzhou University. The patients/participants provided their written informed consent to participate in this study. Written informed consent was obtained from the individual(s) for the publication of any potentially identifiable images or data included in this article.

## Author contributions

YLi and LJ contributed to the data curation. YLiu contributed to the methodology. ZL, BX, and YD contributed to the visualization. YLi contributed to the writing (original draft). YLi and HW contributed to the writing (reviewing and editing). HW contributed to the supervision, resources, and funding acquisition. All authors contributed to the article and approved the submitted version.

## Funding

This research was funded by the Key Research and Development and Promotion projects in Henan province (No. 212102310131). The funders had no role in the study design, data collection and interpretation or the decision to submit the work for publication.

## Conflict of interest

The authors declare that the research was conducted in the absence of any commercial or financial relationships that could be construed as a potential conflict of interest.

## Publisher's note

All claims expressed in this article are solely those of the authors and do not necessarily represent those of their affiliated organizations, or those of the publisher, the editors and the reviewers. Any product that may be evaluated in this article, or claim that may be made by its manufacturer, is not guaranteed or endorsed by the publisher.
