# Supplementary figures and images for: Differences in gastric microbiota and mucosal function between patients with chronic superficial gastritis and intestinal metaplasia
Source: Front Microbiol. 2022 Nov 17;13:950325. doi: 10.3389/fmicb.2022.950325 (PMC9712754; doi:10.3389/fmicb.2022.950325)

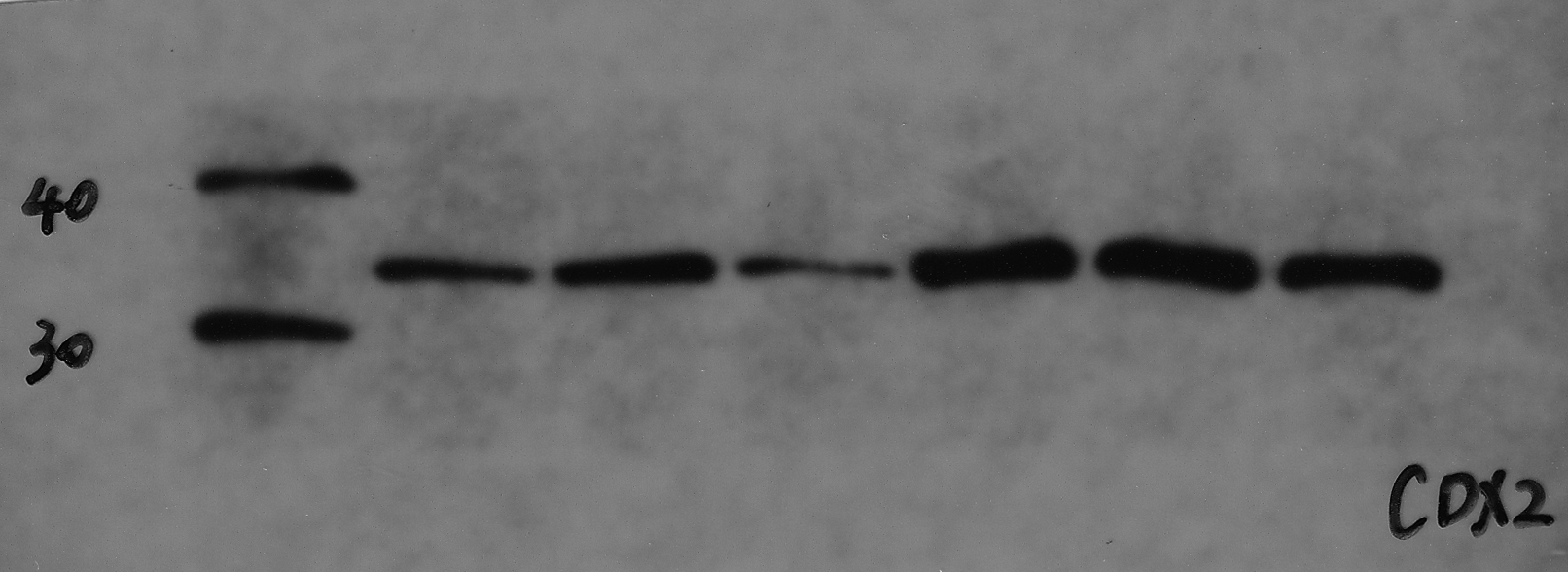

Supplement: Supplementary file 1 [file Data_Sheet_1.zip › Supplementary materials/Original glue map of Fig. 2B/CDX2.jpg]

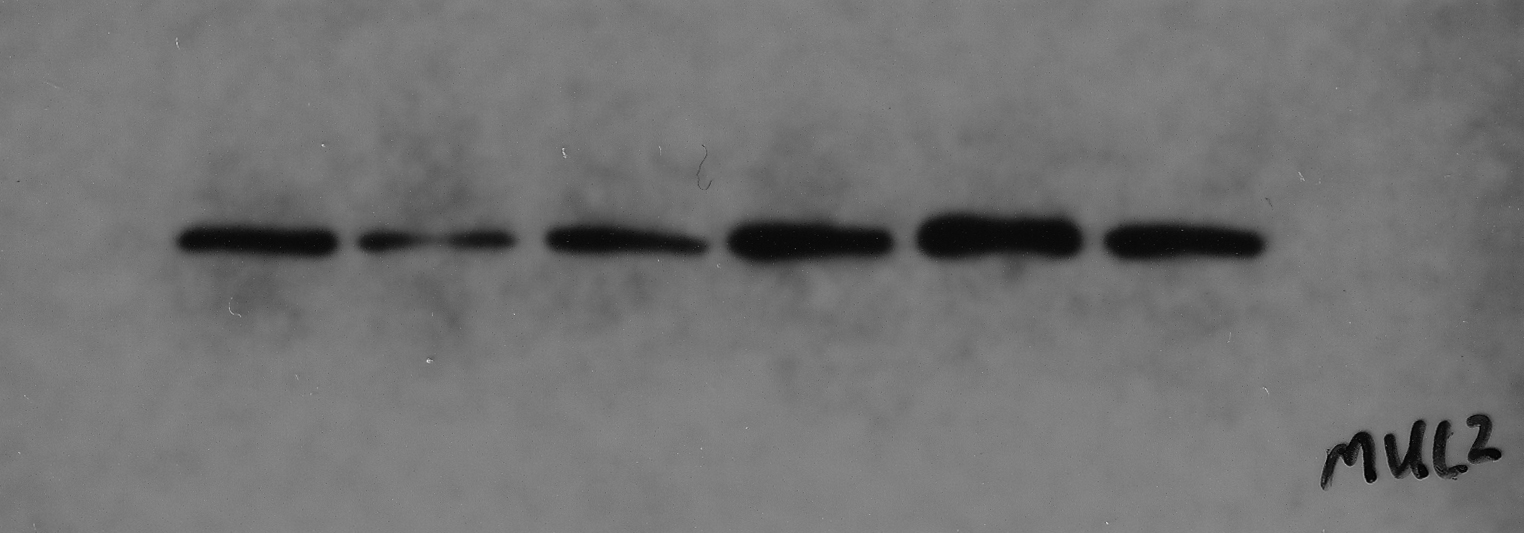

Supplement: Supplementary file 1 [file Data_Sheet_1.zip › Supplementary materials/Original glue map of Fig. 2B/MUC2.jpg]

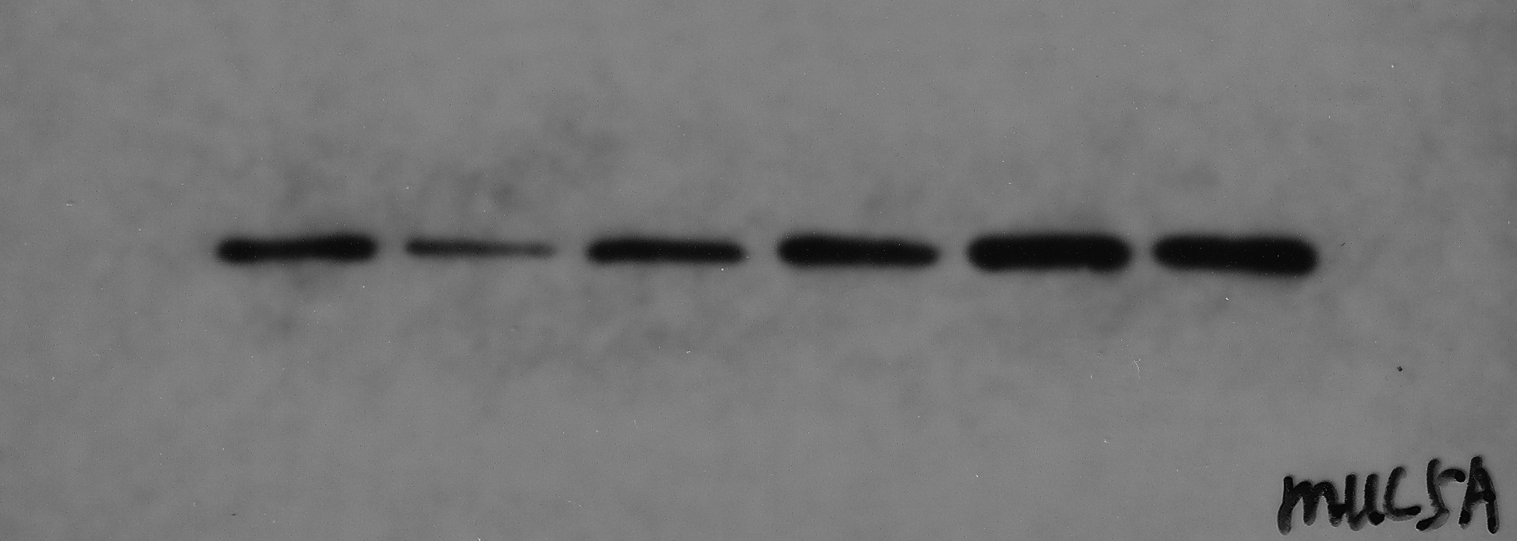

Supplement: Supplementary file 1 [file Data_Sheet_1.zip › Supplementary materials/Original glue map of Fig. 2B/MUC5A.jpg]

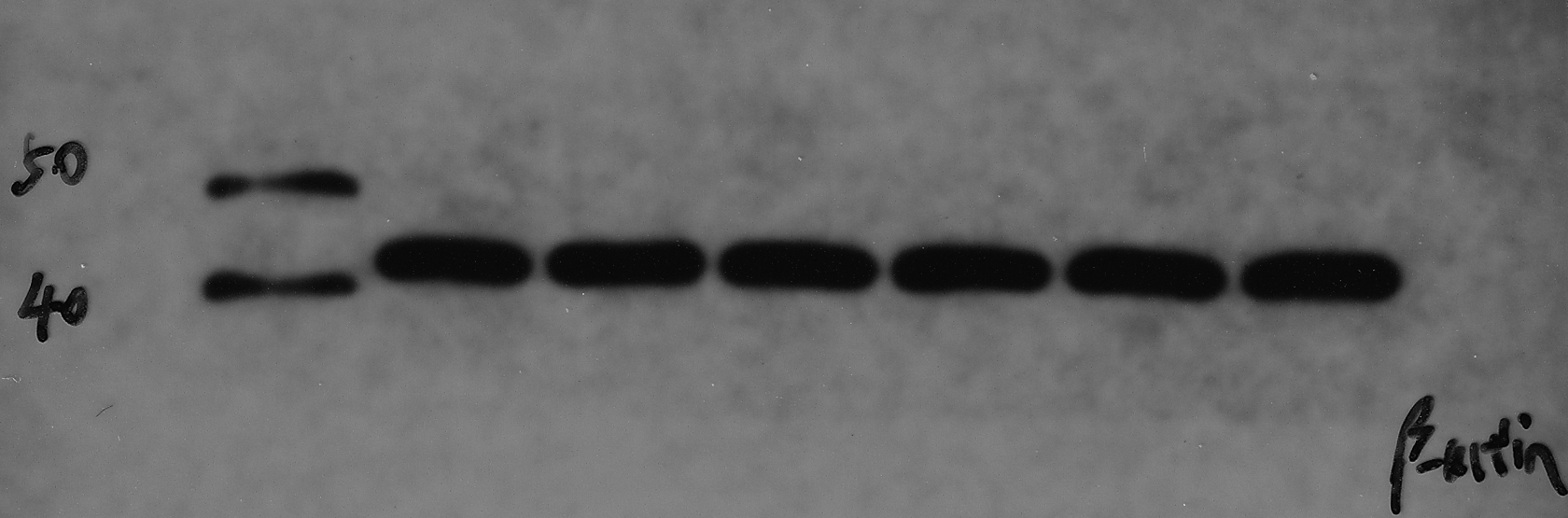

Supplement: Supplementary file 1 [file Data_Sheet_1.zip › Supplementary materials/Original glue map of Fig. 2B/β-actin.jpg]

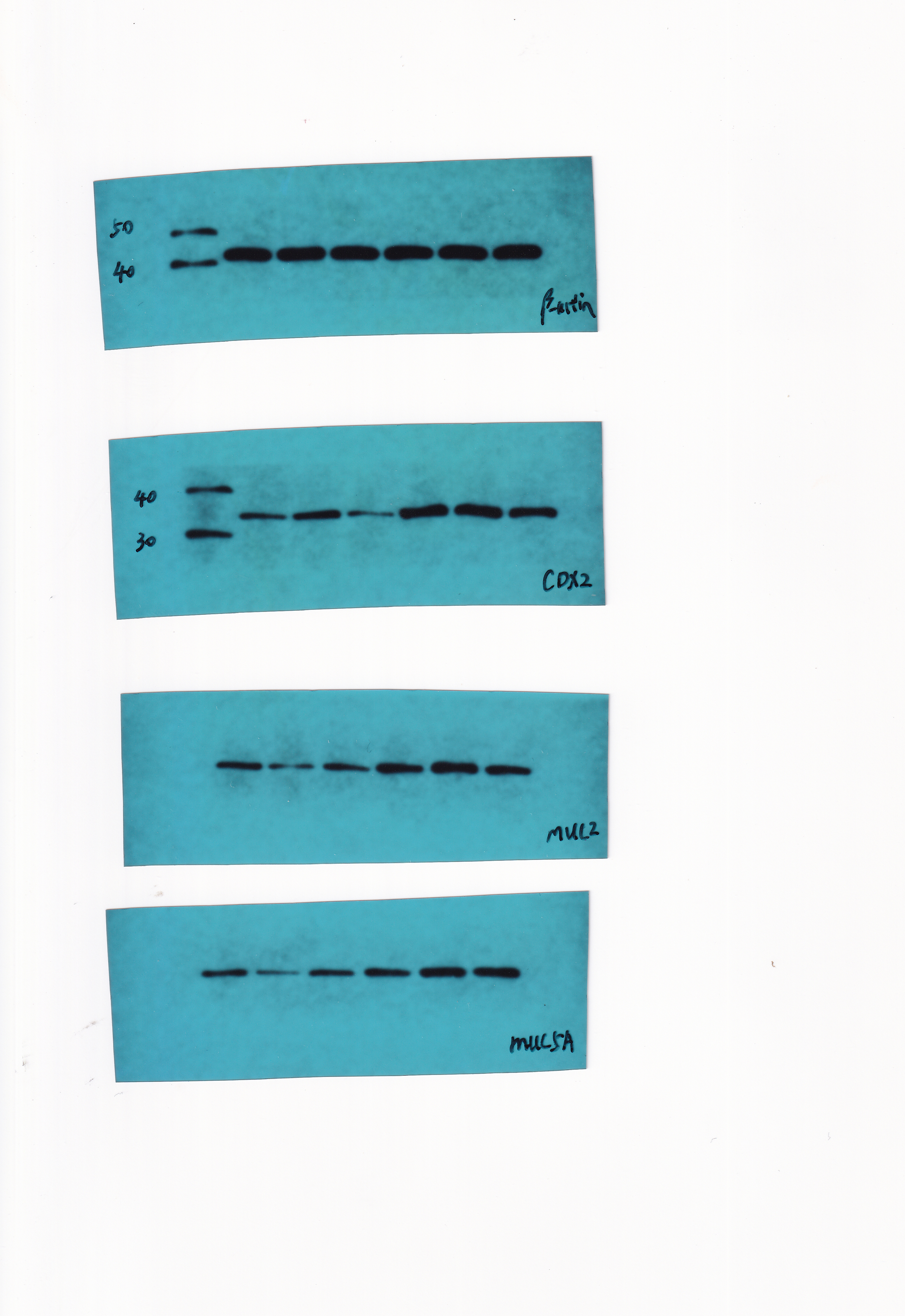

Supplement: Supplementary file 1 [file Data_Sheet_1.zip › Supplementary materials/Original glue map of Fig. 2B/扫描.jpg]
